# Continuation of fibrate therapy in patients with metabolic syndrome and COVID-19: a beneficial regime worth pursuing

**DOI:** 10.1080/07853890.2022.2095667

**Published:** 2022-07-12

**Authors:** Alpo Vuorio, Jonas Brinck, Petri T. Kovanen

**Affiliations:** a Mehiläinen Airport Health Centre, Vantaa, Finland; b Department of Forensic Medicine, University of Helsinki, Helsinki, Finland; c Department of Medicine Huddinge (MEDH7), Karolinska Institutet, Endokrinexpeditionerna C2:94, Karolinska Universitetssjukhuset Huddinge, Stockholm, Sweden; d Unit of Endocrinology, Theme Inflammation and Aging, Karolinska University Hospital Huddinge, Stockholm, Sweden; e Atherosclerosis Research Laboratory, Wihuri Research Institute, Helsinki, Finland

**Keywords:** COVID-19, fibrates, HDL cholesterol, endothelial dysfunction, metabolic syndrome, triglycerides

## Abstract

Based on separate protective mechanisms related to lipid metabolism, viral cell entry and inflammation, fibrate treatment might be advantageous among patients who have been taking fibrates before SARS-CoV-2 infection and continue taking them during the infection. Based on published data on hospitalized COVID-19 patients, we recommend that the clinicians should ask their patients with metabolic syndrome who are already taking fibrates to continue fibrate treatment during the COVID-19 illness. This recommendation applies to both outpatients and hospitalized patients. However, results from the ongoing randomized controlled trials (RCTs) using fenofibrate treatment for the prevention or treatment of COVID-19 have yet to prove that fenofibrate is clinically significant for this indication.

**KEY MESSAGES**
- The role of fibrates as a repurpose to treat SARS-CoV-2 is under investigation in at least three ongoing RCTs.
- Obesity, diabetes, hypertension and dyslipidaemia, individually or clustered as a discrete phenotype, the metabolic syndrome, typically associate with a more severe course of COVID-19.
- Fibrate treatment seems to be most advantageous among patients who have been taken fibrates before SARS-CoV-2 infection and are continuing to take them during the infection.
- We recommend that the clinicians encourage their patients who are already taking fibrate to continue using the drug throughout the COVID-19 illness.

## Introduction

In this research letter, we will briefly address the importance of continuing fibrate treatment in patients with metabolic syndrome who are taking fibrates already before any clinical symptoms of SARS-CoV-2 infection. The role of fibrates as a repurpose to treat SARS-CoV-2 is under investigation in at least three ongoing RCTs [1]. While these RCTs will provide data regarding the potential advantage of fibrates, particularly of fenofibrate, on this indication, it is noteworthy that the infected patients selected for these studies have been fibrate-naïve. It means that we still miss information about the potential role of long-term fibrate use which extends to the period of the actual SARS-CoV-2 infection. Unfortunately, there is the worrying possibility that fibrate administration starting only after a SARS-CoV-2 infection has been detected may be too late. This concern is, at least theoretically, based on the knowledge that fibrates reduce cellular SARS-CoV-2 replication, and thereby could also dampen the immune responses which are driven by viral replication. Accordingly, any supportive pre-emptive treatment with a fibrate could potentially provide clinically significant anti-viral effects during the initial asymptomatic stage of the infection when the patient is still at a low virus load condition [2–5]. This view is supported by research data demonstrating that the concentration of fenofibrate in the lungs is relatively high and reflects that in the blood plasma [6]. Accordingly, we might speculate that fenofibrate could interfere with the production of the lipids needed for the capsid of the coronavirus within the lung tissue, and moreover, that fenofibrate could also interfere with the free fatty acid-binding pocket of the SARS-CoV-2 spike protein, a conformationally regulated functional structure of the receptor-binding domain (RBD) of coronaviruses which has been recently characterized in high detail [7].

## Discussion

More than a decade ago, fenofibrate therapy was shown to increase survival by about 30% in mice infected with the influenza A virus [8]. A recent cell culture study showed that fenofibrate can reverse the metabolic changes induced by SARS-CoV-2 by blocking viral replication [9]. In another recent study carried out in cultured cells by using two different SARS-CoV-2 isolates revealed that both fenofibrate and fenofibric acid (the active metabolite of fenofibrate) were able to reduce cellular SARS-CoV-2 infection by up to 70% [4]. Mechanistically, fenofibric acid destabilized the RBD of the viral spike protein, and so inhibited binding of the virus to its cell surface receptor ACE2 and reduced cellular entry of the virus. Because of the favourable effect applied to two different SARS-CoV-2 isolates, the authors speculated that fenofibrate/fenofibric acid will exert similar antiviral effects on a wide variety of mutations of the spike protein. Importantly, fenofibrate was present in the cell culture medium before the cells were infected by the SARS-CoV-2 isolates. The authors hypothesized that, in addition to reducing the cellular entry of SARS-CoV-2, also other mechanisms of fenofibrate/fenofibric acid, such as a reduction in the inflammatory response and blood clotting could contribute to a potentially favourable effect in patients infected with SARS-CoV-2 [4,10,11].

An observational cross-sectional study of hospitalized COVID-19 patients (*n* = 1411) by Masana et al. examined the clinical significance of varying plasma lipid levels during the infection [12]. The authors studied the lipid profiles available in 1305 patients before hospitalization and in 297 patients during hospitalization. Since SARS-CoV-2 infection itself affects the levels of plasma lipids, the study is of great value, as it compared the lipid values in the two groups of patients both before and during the infection. Interestingly, patients with a severe course of COVID-19 had significantly lower serum high-density lipoprotein (HDL) cholesterol and higher triglyceride levels both before and during hospitalization when compared to those who had a mild course of COVID-19. The authors report that neither body mass index nor the prevalence of diabetes differed between those who had a mild and those who had a severe course of the disease.

Interestingly, in an earlier retrospective study of 413 hospitalized patients of whom 107 (26%) had diabetes, including 21 newly diagnosed diabetes, the newly diagnosed diabetes was a powerful predictor of COVID-19 severity [13]. As discussed by the authors, a significantly adverse effect of hyperglycaemia on COVID-19 outcome was observed in patients with and without in-hospital use of corticosteroids [14]. However, the authors also speculate that the use of glucocorticoids among COVID-19 patients with newly diagnosed diabetes may exacerbate hyperglycaemia and therefore may play a negative role in the outcomes. Thus, in such hospitalized patients, iatrogenic hyperglycaemia may counterbalance the benefits of glucocorticoid therapy, and anti-inflammatory drugs devoid of hyperglycaemic effect may be preferred.

Insulin resistance usually associates with metabolic syndrome and type-2 diabetes, and by downregulating the enzymatic activity of lipoprotein lipase it affects the levels of circulating lipoproteins towards a profile characterized by increased triglycerides and decreased HDL-cholesterol [15]. During infectious and inflammatory states, the cytokines and inflammatory mediators like tumour necrosis factor, interleukin-1, interferon-gamma and lipopolysaccharide, all tend to decrease the activity of the lipoprotein lipase and the level of its regulatory proteins including apo CII, which then results in elevated plasma triglyceride levels and reduced plasma HDL-cholesterol levels [16]. Importantly, such dyslipidaemia can be alleviated by fibrates [17]. In addition to the beneficial effects on dyslipidaemia, i.e. on high triglyceride and low HDL-cholesterol levels, the fibrates show favourable cardiovascular effects which are independent of the beneficial lipid-modulating effects [18,19].

Many underlying comorbidities, such as obesity, diabetes and hypertension have been identified in the progression of COVID-19 into a severe and critical stage, and accordingly, patients with these cardiometabolic conditions have a poorer prognosis than those without them [20]. In a recent multi-country cohort study including 29,040 hospitalized COVID-19 patients with metabolic syndrome, i.e., with a cluster of the above metabolic dysregulations (which included dyslipidaemia), the prognosis was measured by determining whether the patients developed an acute respiratory distress syndrome (ARDS) or died [21]. In the study, the authors tested whether the combined additive or synergistic characteristics of the individual metabolic syndrome-associated comorbidities are independently associated with an increased risk. Importantly, they found that each additional metabolic syndrome criterion was associated with an increased risk of ARDS and death in an additive fashion.

## Conclusion

Based on separate protective mechanisms related to the fibrate effects on the cellular entry of the SARS-CoV-2 virus, inflammation and lipid metabolism, treatment with this drug seems to be most advantageous among patients who have been using fibrates before and during SARS-CoV-2 infection. We recommend that the clinicians inform their patients with metabolic syndrome and an ongoing fibrate treatment that there is no reason to stop taking fibrate during a SARS-CoV-2 infection. Rather, continuing the fibrate treatment is likely to be beneficial. Our recommendation applies to both outpatients and hospitalized patients. The availability of the results from the ongoing RCTs using fenofibrate treatment for a milder SARS-CoV-2 infection and ensuing COVID-19 illness are needed to demonstrate any potential benefit of this drug [22]. Meanwhile, we have to rely on analyses of the current databases of hospitalized COVID-19 patients who have been using fibrates and have or have not continued the treatment during the hospitalization.

## Data Availability

All data relevant to the study are included in the article.
